# Organization and variation analysis of 5S rDNA in gynogenetic offspring of *Carassius auratus red var.* (♀) × *Megalobrama amblycephala* (♂)

**DOI:** 10.1186/s12863-015-0186-z

**Published:** 2015-03-13

**Authors:** QinBo Qin, Juan Wang, YuDe Wang, Yun Liu, ShaoJun Liu

**Affiliations:** Key Laboratory of Protein Chemistry and Developmental Biology of the State Education Ministry of China, College of Life Sciences, Hunan Normal University, Changsha, 410018 People’s Republic of China

**Keywords:** Distant hybridization, Gynogenesis, 5S rDNA, FISH

## Abstract

**Background:**

The offspring with 100 chromosomes (abbreviated as GRCC) have been obtained in the first generation of *Caras*sius *auratus red var.* (abbreviated as RCC, 2n = 100) (♀) × *Megalobrama amblycephala* (abbreviated as BSB, 2n = 48) (♂), in which the females and unexpected males both are found. Chromosomal and karyotypic analysis has been reported in GRCC which gynogenesis origin has been suggested, but lack genetic evidence.

**Result:**

Fluorescence *in situ* hybridization with species-specific centromere probes directly proves that GRCC possess two sets of RCC-derived chromosomes. Sequence analysis of the coding region (5S) and adjacent nontranscribed spacer (abbreviated as NTS) reveals that three types of 5S rDNA class (class I; class II and class III) in GRCC are completely inherited from their female parent (RCC), and show obvious base variations and insertions-deletions. Fluorescence in situ hybridization with the entire 5S rDNA probe reveals obvious chromosomal loci (class I and class II) variation in GRCC.

**Conclusions:**

This paper provides directly genetic evidence that GRCC is gynogenesis origin. In addition, our result is also reveals that distant hybridization inducing gynogenesis can lead to sequence and partial chromosomal loci of 5S rDNA gene obvious variation.

## Background

Gynogenesis is an important reproductive mode in fish. The Amazon Molly, *Poecilia formosa*, is one such fish that reproduces by gynogenesis and produces all-female offspring. Natural gynogenetic males of this species are, however, also found and several studies have documented that they are of hybrid origin [1]. UV-treated sterile sperm of *Megalobrama amblycephala* (BSB, 2n = 48) and cold shock (0–4°C) for 30 min to double the eggs’ chromosomes have been used to activate *Caras*sius auratus red var. (RCC, 2n = 100) eggs, resulting in all-female gynogenetic progeny [2]. Interestingly, we obtained both male and female offspring with 100 chromosomes (GRCC) in the first generation of *Carassius auratus red var.* (RCC, 2n = 100) (♀) × *Megalobrama amblycephala* (BSB, 2n = 48) (♂). Chromosomal and karyotypic analysis has been reported in GRCC which gynogenesis origin has been suggested referring to it’s the female parent [3], but lack sufficient genetic evidence.

In vertebrate, 5S rDNA consists of a conserved coding region of 120 bp (5S) with a variable intergenic spacer usually referred to as the nontranscribed spacer (NTS) [4-7]. The coding region is highly conserved, even among nonrelated taxa, whereas the spacer region is more variable. Many studies have shown that birth-and-death processes and selection can drive the evolution of 5S rDNA in distantly related taxa [8-11]. Thus, 5S rDNA sequences have been used as genetic and cytogenetic markers in evolutionary studies and for studying intraspecific variation, genome evolution, and phylogenetic reconstruction [12-15]. Indeed, little is known about the nucleotide sequences and chromosomal location of 5S rRNAs in teleosts in comparison with other vertebrate classes. Our previous study revealed the influence of polyploidy on the organization and evolution of the 5S rDNA multigene family in teleosts [16]. In an attempt to further understand the genetic variation in gynogenetic fish, and to extend the knowledge of 5S rDNA organization in teleosts, the 5S rDNA sequences and chromosomal loci of RCC and GRCC were isolated and characterized. Our data reveal the genetic variation and evolutionary characteristics of the 5S rDNA multigene family in fish, and provide new insights into the evolutionary patterns of this multigene family in vertebrates.

## Methods

All samples were cultured in ponds at the Protection Station of Polyploidy Fish, Hunan Normal University, and fed with artificial feed. Fish treatments were carried out according to the regulations for protected wildlife and the Administration of Affairs Concerning Animal Experimentation, and approved by the Science and Technology Bureau of China. Approval from the Department of Wildlife Administration was not required for the experiments conducted in this paper. The fish were deeply anesthetized with 100 mg/L MS-222 (Sigma-Aldrich, St Louis, MO, USA) before dissection.

### Crosses

During the reproductive seasons (from April to June) in 2006 and 2007, each 15 mature females and 15 mature males of both RCC and BSB were chosen as the maternal fish and paternal fish, respectively. The crossings were performed by two groups. In the first group, RCC was used as the maternal parent, and BSB was used as the paternal fish. In the second one, the maternal fish and paternal fish were reversed. In the reverse cross-BSB(♀) × RCC(♂), there was no living progeny, while in the cross-RCC(♀) × BSB(♂), there existed the living offspring including diploid gynogenetic red crucian carp (GRCC), triploid and tetraploid hybrids [3].

### Preparation of chromosome spreads

To determine ploidy, chromosome counts were performed using kidney tissue from 20 individuals each of BSB, RCC, and GRCC at 1 year of age. After culture for 1–3 d at a water temperature of 18–22°C, the samples were injected with concanavalin one to three times at a dose of 2–8 mg/g body weight. The interval between injections was 12–24 h. Six hours prior to dissection each sample was injected with colchicine at a dose of 2–4 mg/g body weight. The kidney tissue was ground in 0.9% NaCl, followed by hypotonic treatment with 0.075 m KCl at 37°C for 40–60 min and then fixed in 3:1 methanol–acetic acid with three changes. The cells were dropped onto cold, wet slides and stained for 30 min in 4% Giemsa. The shape and number of chromosomes were analyzed under a microscope. For each type of fish, 400 metaphase spreads (20 metaphase spreads from each sample) of chromosomes were analyzed. The preparations were examined under an oil lens at a magnification of 3330 ×.

### PCR amplification and sequencing of 5S rDNA sequences

Total genomic DNA was isolated from peripheral blood cells according to the standard phenol: chloroform extraction procedure described Sambrook et al. [17]. One pair of primers (5SP1, 5′-GCTATGCCCGATCTCGTCTGA-3′: 5SP2R, 5′-CAGGTTGGTATGGCCGTAAGC-3′) was designed and synthesized to PCR amplify 5S rDNA repeats directly from genomic DNA. The PCR reaction was performed in a volume of 25 μl with approximately 20 ng of genomic DNA, 1.5 mM of MgCl_2_, 200 μM of each dNTP, 0.4 μM of each primer, and 1.25 U of Taq polymerase (Takara). The temperature profile was: initial denaturation step at 94°C for 5 min, followed by 30 cycles of 94°C for 30 s, 56°C for 30 s, and 72°C for 1 min, with a final extension step at 72°C for 10 min. Amplification products were separated on a 3.0% agarose gel using TBE buffer. The DNA fragments were purified using a gel extraction kit (Sangon) and ligated into pMD-18 T. Plasmids were transformed into *Escherichia coli* DH5a, propagated and then purified. The cloned DNA fragments were sequenced by an automated DNA sequencer (ABI PRISM 3730). Sequence homology and variation among the fragments amplified from RCC and GRCC were analyzed using ClustalW software (http://www.ebi.ac.uk/clustalw/ intex.html).

### Fluorescence *in situ* hybridization

The probes of fluorescence in situ hybridization (FISH) for 5S gene and Species-specific centromere were constructed for RCC and amplified by PCR using the 5SP1 and 5SP2R primers, the primers 5′-TTCGAAAAGAGAGAATAATCTA-3′ and 5′-AACTCGTCTAAACCCGAACTA-3′, respectively. The PCR reaction of Species-specific centromere primers was performed in a volume of 25 μl with approximately 20 ng of genomic DNA, 1.5 mM of MgCl_2_, 200 μM of each dNTP, 0.4 μM of each primer, and 1.25 U of Taq polymerase (Takara). The temperature profile was: initial denaturation step at 94°C for 5 min, followed by 35 cycles of 94°C for 30 s, 58°C for 30 s, and 72°C for 1 min, with a final extension step at 72°C for 10 min. The FISH probes were produced by Dig-11-dUTP labeling (using a nick translation kit, Roche, Germany) of purified PCR products. FISH was performed according to the method described by Caradonna et al. [18]. For each type of fish, 200 metaphase spreads with 100 chromosomes (20 metaphase spreads in each sample) were analyzed.

## Results

### Chromosome number and composition

**Figure 1:**
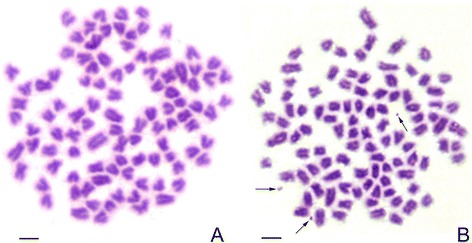
**Chromosome spreads at metaphase in RCC and GRCC. A**: The 100 chromosome of RCC; **B**: The 100 chromosomes and three micro-chromosomes (arrow) of GRCC; bar = 3 μm.

In RCC, 90.3% of metaphases showed 100 chromosomes without microchromosomes (Figure 1A; Table 1). In GRCC, 93.3% of metaphases possessed 100 chromosomes with one to three microchromosomes (Figure 1B; Table 1).

**Table 1:** **Examination of chromosome number of RCC and GRCC**

**Fish type**	**No. of fish**	**No. of metaphase**	**Distribution of chromosome number**	
			**<100**	**100**
RCC	20	400	39	361
GRCC	20	400	27	373

**Figure 2:**
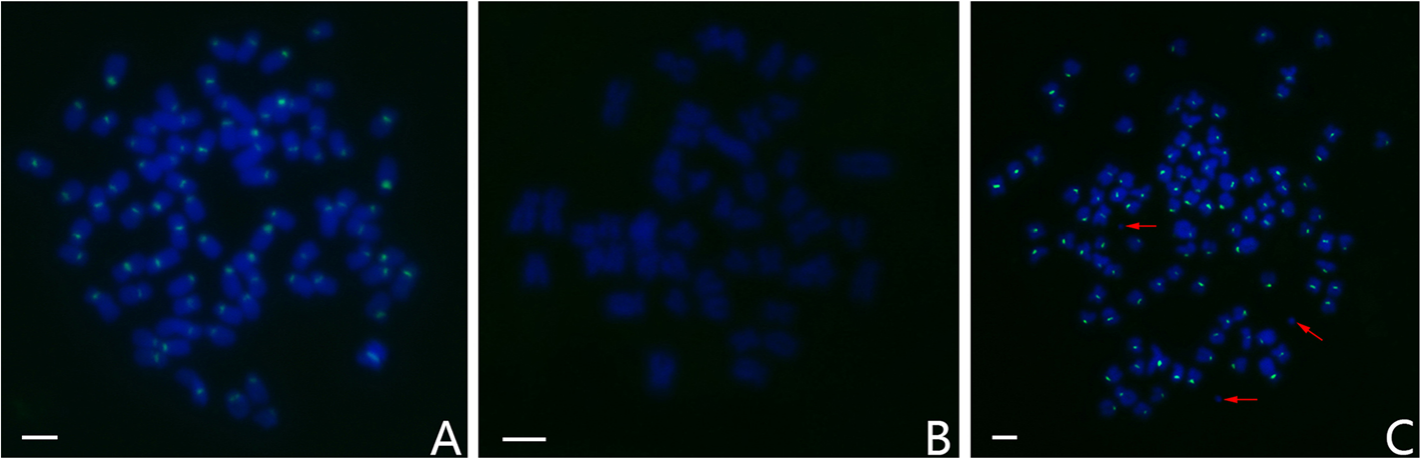
**Examination of hybridizing signals by FISH (species-specific centromere probe) in RCC, BSB and GRCC. A**: The centromere probe hybridized to 100 chromosomes in RCC; **B**: No chromosomes of BSB were hybridized; **C**: The centromere probe hybridized to 100 chromosomes of GRCC, red arrows indicate micro-chromosomes; bar = 3 μm.

The species-specific centromere probe (sequence number: JQ086761) hybridized to 100 chromosomes of RCC (Figure 2A), whereas no chromosomes of BSB was hybridized (Figure 2B). Thus, RCC and BSB-derived chromosomes can be discriminated by FISH using the centromere probe. As expected, the centromere probe hybridized to 100 chromosomes of GRCC (Figure 2C), suggesting that they possess two sets of RCC-derived chromosomes and were diploid gynogenetic fish.

### 5S rDNA sequence characterization and molecular analysis

**Figure 3:**
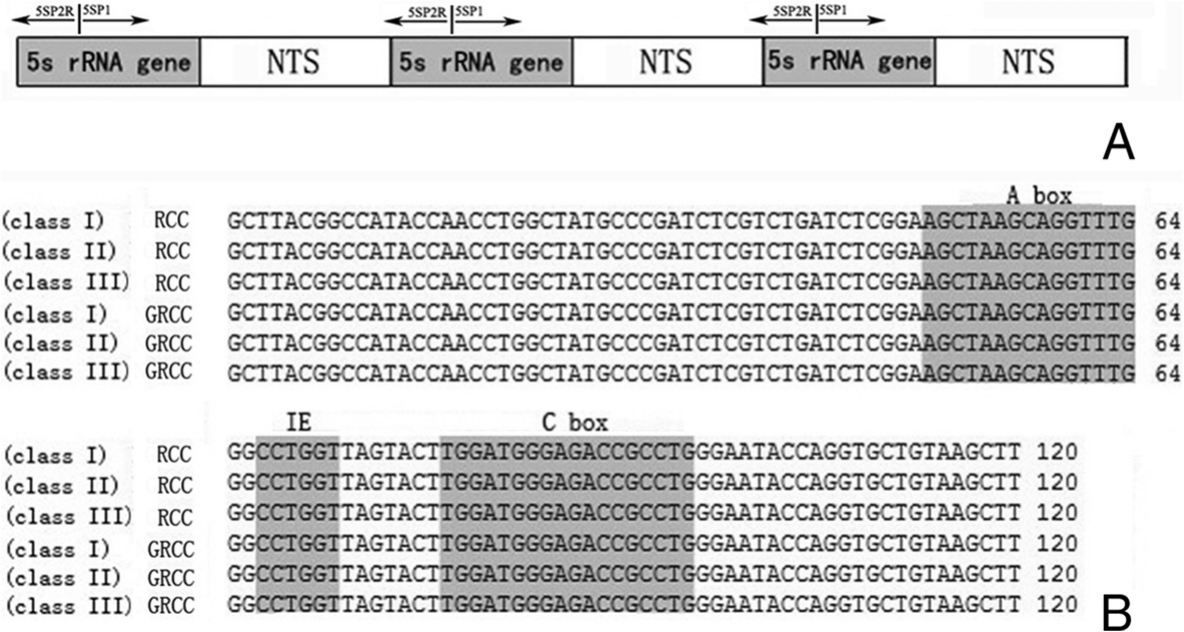
**Representative sequences of monomeric 5S rDNA. A**: Arrangement of higher eukaryotic 5S rRNA genes intercalated with nontranscribed DNA segments (NTS); **B**: Complete 5S coding regions from RCC and GRCC, nucleotide variation was not detected, internal control regions of the coding region are shaded.

PCR amplification of segments containing 5S rDNA from all specimens using the primers 5SP1 and 5SP2R generated fragments of approximately 200, 340, and 500 bp from RCC and GRCC. All the cloned sequences contained a 120-bp coding sequence of the 5S ribosomal gene and NTS regions of different length (Figure 3A). In RCC, the three types of 5S rDNA classes (designated class I: 203 bp; class II: 340 bp; and class III: 477 bp) were characterized by distinct NTS types (designated NTS-I, NTS-II and NTS-III for the 83-, 220- and 357-bp sequences, respectively). GRCC had three types of 5S rDNA classes, which were completely inherited from RCC (class I, class II and class III). All 5S rDNA sequences have been submitted to GenBank, and their accession numbers are listed in Table 2.

**Table 2:** **GenBank accession numbers of the 5S rDNA sequences in RCC and GRCC**

**DNA fragments**	**GenBank accession no. of the sequences**	
	**RCC**	**GRCC**
203 bp	GQ485555	JX876906
338 bp;340 bp	GQ485556	JX876907
477 bp;487 bp	GQ485557	JX876908

**Figure 4:**
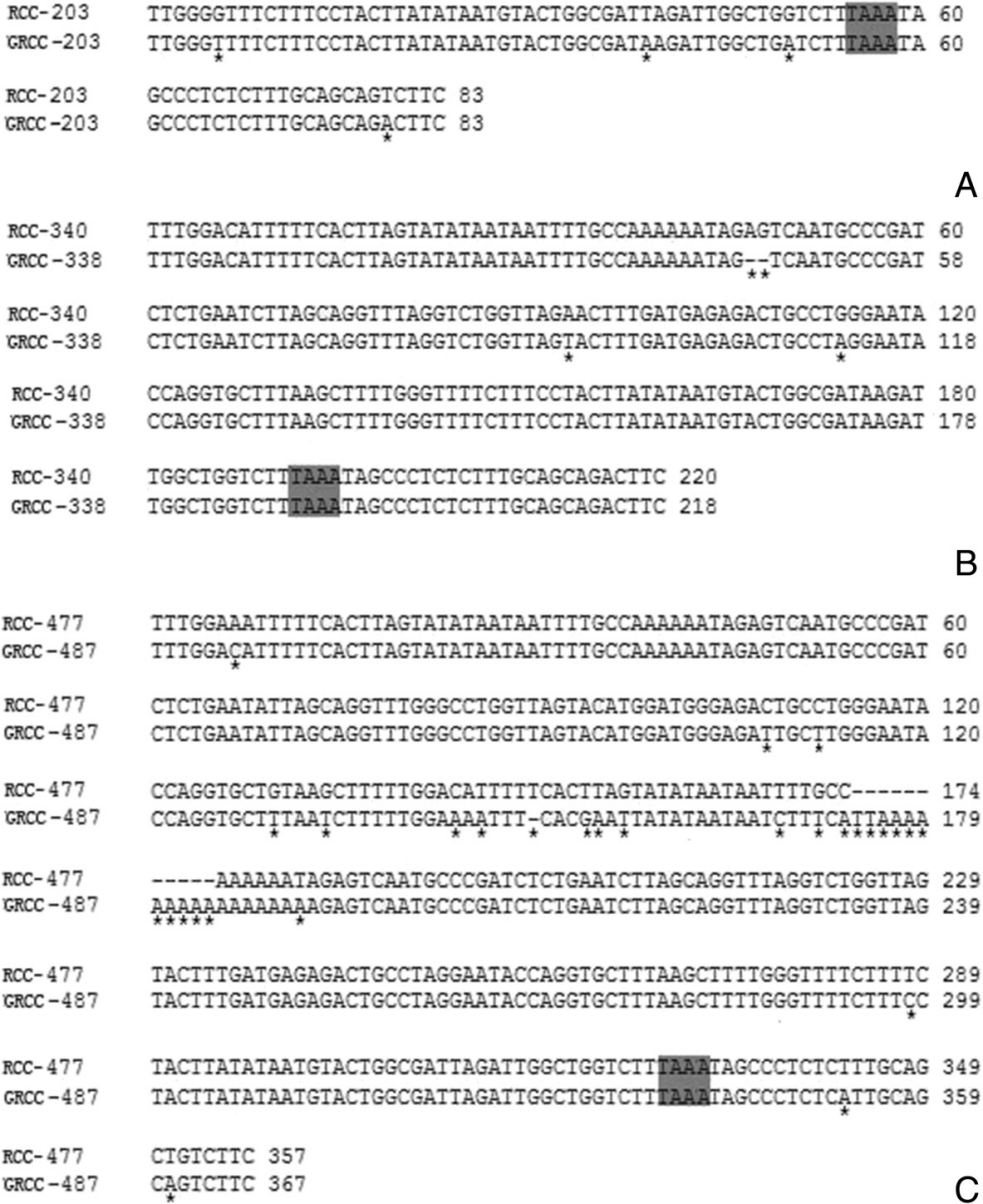
**Comparison of the NTS sequences from RCC and GRCC. A**: the NTS-I from RCC and GRCC; **B**: the NTS-II from RCC and GRCC; **C**: the NTS-III from RCC and GRCC; asterisks mark variable sits in NTS; The NTS upstream TATA elements are shaded.

RCC had three types of 5S rDNA with the same coding region but distinct NTS sequences. As expected, comparison of the 120-bp coding region of RCC 5S rDNA with those of RCC and GRCC revealed great similarity (Figure 3B). Among the internal control regions (ICRs, i.e. the promoters for transcription), nucleotide variation was not detected in GRCC. A comparison of NTS-I revealed four base substitutions among the sequences (Figure 4A). A comparison of NTS-II showed two base substitutions and a deletion-insertion at position −174 (Figure 4B). A comparison of NTS-III elements showed 16 base substitutions and a deletion-insertion at position −183 and −150 (Figure 4C). In addition, characterization of the NTS-upstream region showed that TATA control element, the regulatory region for 5S gene transcription, which was identifiable in the NTS of RCC and GRCC (at 29 in all NTS sequences, where it was modified to TAAA) (Figure 4).

### Chromosomal loci of 5S rDNA

**Figure 5:**
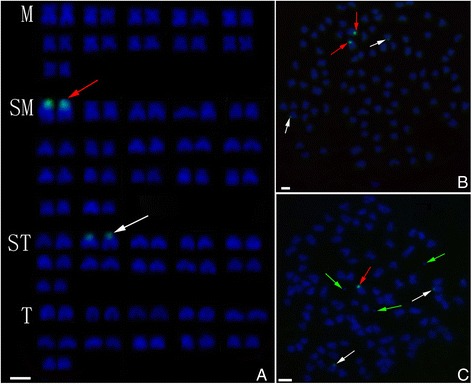
**Examination of hybridizing signals by FISH (class II) in RCC and GRCC. A**: The karyotype of RCC indicated that a pair of big 5S gene loci (class II) was located on a homologous submetacentric chromosome (red arrows), and a pair of small 5S gene loci was located on a homologous subtelocentric chromosome (white arrows); **B**: There were 2 big (red arrows) and 2 small 5S gene loci (white arrows) (class II) in RCC; **C**: There were 1 big (red arrows) and 2 small 5S gene loci (white arrows) (class II) in GRCC, green arrows indicate micro-chromosomes; bar = 3 μm.

**Figure 6:**
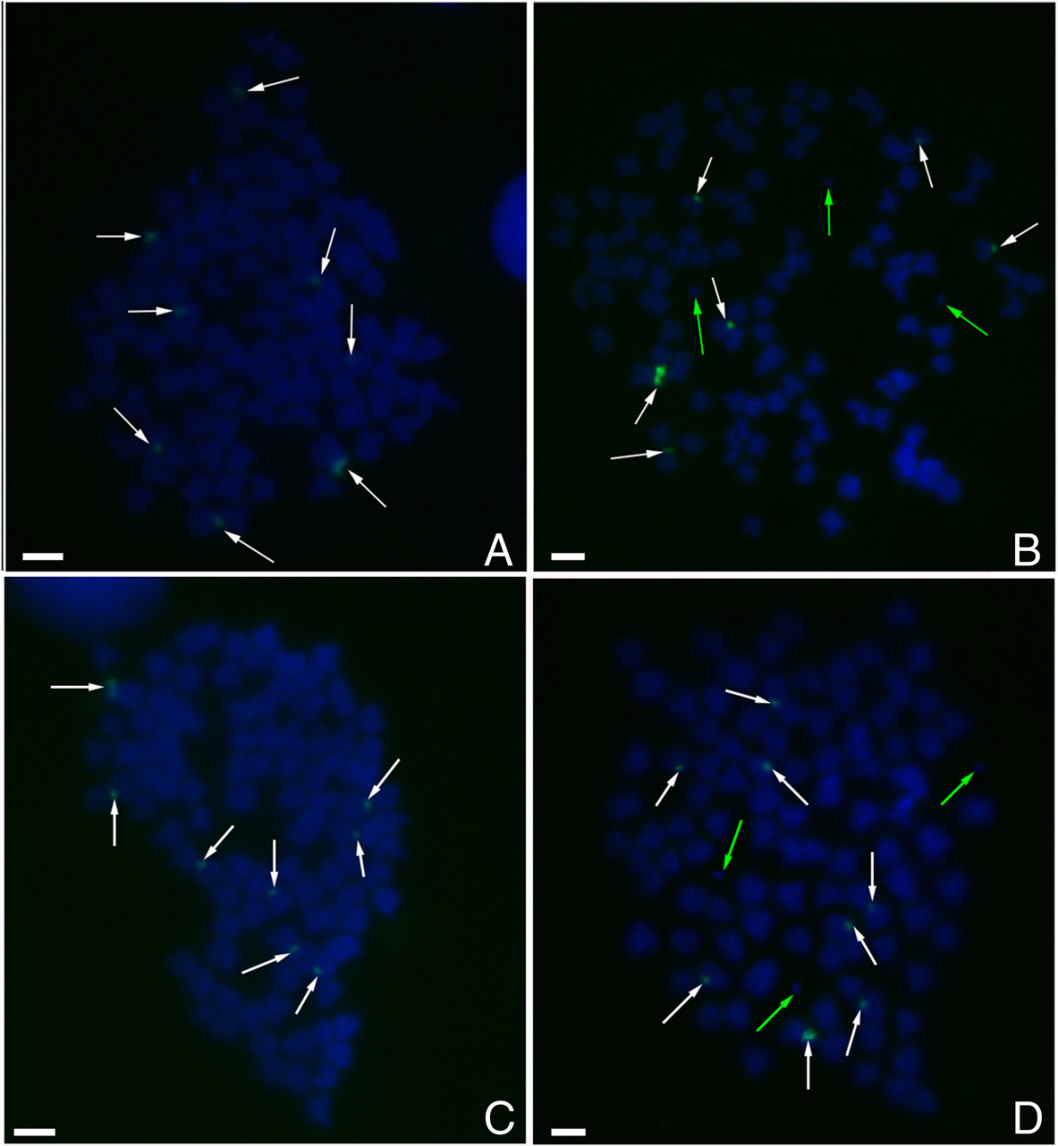
**Examination of hybridizing signals by FISH (class I and class III) in RCC and GRCC. A**: There were 8 hybridizing signals (white arrows) (class I) in RCC; **B**: There were 6 hybridizing signals (white arrows) (class I) in GRCC, three microchromosomes (green arrows) were also observed; **C**: There were 8 hybridizing signals (white arrows) (class III) in RCC; **D**: There were 8 hybridizing signals (white arrows) (class III) in GRCC, three microchromosomes (green arrows) were also observed; bar = 3 μm.

5S rDNA fluorescent probes were prepared from cloned 5S rDNA repeated units from RCC (class II and class I), and the results of FISH are showed in Table 3. The probe was clearly detected in the pericentromeric region of chromosomes in RCC and GRCC (Figure 5). The hybridization of 5S rDNA (class II) probes showed two large 5S gene loci and two small loci in 86% RCC chromosomal metaphases (Figure 5B). Chromosomal locus map revealed that two large 5S gene loci were located on a homologous submetacentric chromosome, and two small 5S gene loci were located on a homologous subtelocentric chromosome (Figure 5A; Figure 5B). Two large and two small 5S gene loci were also expected in GRCC, however, only one large and two small 5S gene loci were detected in 88% GRCC chromosomal metaphases (Figure 5C). A large 5S gene locus on the submetacentric chromosome was deleted. The hybridization of 5S rDNA (class I) probes showed eight 5S gene loci in 82% RCC chromosomal metaphases (Figure 6A). Eight 5S loci were also expected in GRCC, but only six 5S loci were found in 84.5% GRCC chromosomal metaphases (Figure 6B). The hybridization of 5S rDNA (class III) probes showed eight 5S gene loci in 93% RCC chromosomal metaphases (Figure 6C). Eight 5S loci were also found in 88.5% GRCC chromosomal metaphases (Figure 6D). These results suggested that partial chromosomal loci of 5S rDNA were variation in GRCC.

**Table 3:** **Examination of chromosomal locus in RCC and GRCC**

**Fish type**	**No. of chromosome**	**The probe with 203 bp**	**The probe with 477 bp**	**The probe with 340 bp**	
		**No. of locus**	**No. of locus**	**No. of large locus**	**No. of small locus**
RCC	100	8	8	2	2
GRCC	100	6	8	1	2

## Discussion

Gynogenesis is an especial reproductive mode, in which genetic material of the female parent controls individual development. The main differences between natural gynogenesis and artificial gynogenesis are the UV treatment of sperm and the cold shock to eggs. BSB sperm without UV treatment were able to enter RCC eggs, but BSB genetic material is excluded during embryonic development. Thus, GRCC possess two sets of RCC-derived chromosome and BSB-derived microchromosomes [3]. This study also provides directly genetic evidence that GRCC is gynogenesis origin.

Our previous study revealed the influence of polyploidy on 5S rDNA in fish, including parental genome-specific loss, formation of new types of 5S rDNA class, substitutions and insertion-deletions in the NTS sequence [16]. The evolution of 5S rDNA is driven by birth-and-death processes with strong purifying selection [8-11], which can lead to the existence of the different types of NTS [10]. In this paper, GRCC have three types of 5S rDNA class that completely derived from their female parent (RCC), but no new types of 5S rDNA class is found. Although obvious base variation and insertions-deletions of NTS of 5S rDNA were also observed in GRCC, all sequences analyzed here were likely to correspond to functional genes, because they exhibit all the necessary features for the correct gene expression: three ICRs (box A, internal element, and box C), a TATA control element, and a T-rich tail. It was possible for the genome of the BSB sperm to recombine with the genome of RCC, which could lead to genetic variation via recombination, for example, *sox* gene shows obvirous variation in GRCC [3]. In this article, our results revealed that GRCC had three types of NTS sequences, in which NTS-III showed an insertion-deletion and obvious nucleotide variations. Thus, we confirmed that the formation of bisexual gynogenetic fish by hybridization can induce obvious base substitutions and insertion-deletions in the NTS sequence, and that these variations in the NTS sequence can serve as an effective molecular marker to distinguish RCC and GRCC.

Fluorescence in-situ hybridization (FISH) is effective way to determine the chromosomal location of the 5S rDNA. Using specific NTS sequences as probes is able to check the exact chromosome location of the different 5S rDNA classes. In this paper, in addition to the 5S ribosomal gene characterization among RCC and GRCC, the chromosomal loci of 5S rDNA performed here allows for a more thorough cytogenetic comparison. FISH, using the 5S rDNA sequence with 340 bp (class II) as probe, showed that a pair of large 5S gene loci were located on a homologous submetacentric chromosome, and a pair of small 5S gene loci were located on a homologous subtelocentric chromosome**.** Two large and two small 5S gene loci were expected in GRCC, however, only one large and two small 5S gene loci were detected. A large 5S gene locus on the submetacentric chromosome was deleted, suggesting that the chromosomal location of the 340 bp 5S rDNA was changed in GRCC. In addition, hybridization of 5S rDNA sequence with 203 bp (class I) to RCC metaphase chromosomes showed eight 5S gene loci. Eight 5S loci were expected in GRCC, but only six 5S loci were found. It is also reveals chromosomal loci variation in GRCC.

Several studies have documented that such changes to the genome are prerequisites for the successful establishment of a newly formed hybrid or allopolyploid species, because the purging of genetic incompatibility is essential for high fertility in a hybrid or allopolyploid that has diverged from the parents [19-22]. For example, a positive correlation between the amount and rapidity of sequence elimination and the fertility of the various newly synthesized allopolyploids has been observed [23]. GRCC metaphase spreads possess two sets of RCC-derived chromosome and BSB-derived microchromosome [3]. We hypothesized that, to improve fertility, GRCC are required for genetic recombination, mutation, and removal of DNA to reduce incompatible parental genetic material (RCC and BSB). Consequently, recombination and elimination of 5S rDNA sequences and Chromosomal loci variation were revealed in GRCC.

## Conclusions

We obtained both male and female offspring with 100 chromosomes (GRCC) in the first generation of *Carassius auratus red var.* (RCC, 2n = 100) (♀) × *Megalobrama amblycephala* (BSB, 2n = 48) (♂) [3]. This paper provides directly genetic evidence that GRCC is gynogenesis origin. In addition, our results also prove that distant hybridization inducing gynogenesis can lead to sequence and partial chromosomal loci of 5S rDNA gene obvious variation.

## References

[1] Hubbs C, Drewry GE, Warburton B (1959). Occurrence and morphology of a phenotypic male of a gynogenetic fish. Science.

[2] Sun YD, Tao M, Liu SJ, Zhang C, Duan W, Shen JM (2007). Induction of gynogenesis in red crucian carp using spermatozoa of blunt snout bream. Prog Nat Sci.

[3] Liu SJ, Qin QB, Wang YQ, Zhang H, Zhao RR, Zhang C (2010). Evidence for the formation of the male gynogenetic fish. Mar Biotechnol.

[4] Korn LJ, Brown DD (1978). Nucleotide sequence of Xenopus borealis oocyte 5S DNA: comparison of sequences that flank several related eucaryotic genes. Cell.

[5] Nielsen JN, Hallenberg C, Frederiksen S, Sørensen PD, Lomholt B (1993). Transcription of human 5S rRNA genes is influenced by an upstream DNA sequence. Nucleic Acids Res.

[6] Hallenberg C, Frederiksen S (2001). Effect of mutations in the upstream promoter on the transcription of human 5S rRNA genes. Biochim Biophys Acta-Gene Struc Expr.

[7] Pasolini P, Costagliola D, Rocco L, Tinti F (2006). Molecular organization of 5S rDNAs in Rajidae (Chondrichthyes): structural features and evolution of piscine 5S rRNA genes and nontranscribed intergenic spacers. J Mol Evol.

[8] Nei M, Rooney AP (2005). Concerted and birth-and-death evolution of multigene families. Annu Rev Genet.

[9] Perina A, Seoane D, González-Tizón AM, Rodríguez-Fariña F, Martínez-Lage A (2011). Molecular organization and phylogenetic analysis of 5S rDNA in crustaceans of the genus Pollicipes reveal birth-and-death evolution and strong purifying selection. BMC Evol Biol.

[10] Pinhal D, Yoshimura TS, Araki CS, Martins C (2011). The 5S rDNA family evolves through concerted and birth-and-death evolution in fish genomes: an example from freshwater stingrays. BMC Evol Biol.

[11] Vizoso M, Vierna J, González-Tizón AM, Martínez-Lage A (2011). The 5S rDNA gene family in mollusks: characterization of transcriptional regulatory regions, prediction of secondary structures, and long-term evolution, with special attention to Mytilidae mussels. J Hered.

[12] Suzuki H, Moriwaki K, Sakurai S (1994). Sequences and evolutionary analysis of mouse 5S rDNAs. Mol Biol Evol.

[13] Pendas AM, Moran P, Martinez JL, Garcia-Vazquez E (1995). Applications of 5S rDNA in Atlantic salmon, brown trout, and in Atlantic salmon brown trout hybrid identification. Mol Ecol.

[14] Sajdak SL, Reed KM, Phillips RB (1998). Intraindividual and interspecies variation in the 5S rDNA of coregonid fish. J Mol Evol.

[15] Martins C, Galetti P (2001). Two 5S rDNA arrays in Neotropical fish species: is it a general rule for fishes?. Genetica.

[16] Qin QB, He WG, Liu SJ, Wang J, Xiao J, Liu Y (2010). Analysis of 5S rDNA organization and variation in polyploid hybrids from crosses of different fish subfamilies. J Exp Zool B Mol Dev Evol.

[17] Sambrook J, Fritsch E, Maniatis T (1989). Molecular cloning: a laboratory manual.

[18] Caradonna F, Bellavia D, Clemente AM, Sisino G, Barbieri R (2007). Chromosomal localization and molecular characterization of three different 5S ribosomal DNA clusters in the sea urchin Paracentrotus lividus. Genome.

[19] Rieseberg LH (2001). Polyploid evolution: keeping the peace at genomic reunions. Curr Biol.

[20] Kashkush K, Feldman M, Levy AA (2002). Gene loss, silencing and activation in a newly synthesized wheat allotetraploid. Genetics.

[21] Kotseruba V, Gernand D, Meister A, Houben A (2003). Uniparental loss of ribosomal DNA in the allotetraploid grass Zingeria trichopoda (2 n = 8). Genome.

[22] Han F, Fedak G, Guo W, Liu B (2005). Rapid and repeatable elimination of a parental genome-specific DNA repeat (pGc1R-1a) in newly synthesized wheat allopolyploids. Genetics.

[23] Ozkan H, Levy AA, Feldman M (2001). Allopolyploidy-induced rapid genome evolution in the wheat (Aegilops–Triticum) group. Plant Cell Online.

